# Efficacy, Safety, and Tolerance of Split Dose Oral Sulfate Solution Versus Split-Dose Polyethylene Glycol Versus Single Dose Polyethylene Glycol for Colonoscopy Preparation: A Prospective Randomized Study

**DOI:** 10.34172/mejdd.2025.403

**Published:** 2025-01-31

**Authors:** George Sarin Zacharia, Varghese Thomas

**Affiliations:** ^1^BronxCare Health System, Bronx, USA & Ahalia Hospital, Abu Dhabi, UAE; ^2^Malabar Medical College Hospital and Research Center, Calicut, Kerala, India

**Keywords:** Colonoscopy, Split dose bowel preparation, PEG, Oral sulfate solution, OSS, BBPS

## Abstract

**Background:**

The quality of bowel preparation is one of the key determinants of a successful colonoscopy. Bowel preparation regimens have evolved greatly over the past few decades, with attempts to improve the efficiency and tolerability; still an ideal agent or regimen continues to be oblivious. To compare the efficacy, safety, and tolerance of three bowel preparation regimens for colonoscopy: split dose of oral sulfate solution (OSS), split dose of polyethylene glycol (PEG), and same-day single dose PEG.

**Methods:**

This study was a randomized, single-blind control design with three study groups. Group A received a split dose of OSS, group B received a split dose of PEG, and Group C received a single dose of PEG for bowel preparation. The quality of preparation was assessed using the Boston Bowel Preparation Scale (BBPS), and the adverse effects and tolerance were noted. The data were compared statistically for any significant difference between the regimens.

**Results:**

Mean total BBPS scores were 8.08, 7.52, and 7.92 for groups A, B, and C, respectively (*P*=0.076). Segmental BBPS scores were statistically similar for the right and transverse colon but differed for the left colon (A: B: C=2.79: 2.54: 2.75; *P*<0.01). Gastrointestinal side effects and electrolyte disturbances were similar across the three groups. Split-dose preparations were associated with more significant sleep disturbances than single-dose PEG (*P*<0.001). Patients who received OSS reported more taste intolerance (*P*<0.01), while those who received single PEG reported more volume intolerance (*P*<0.001).

**Conclusion:**

Split-dose OSS, split-dose PEG, and single-dose PEG regimens provide adequate and comparable bowel preparation for colonoscopy with good patient tolerance and no significant adverse effects. Overnight PEG and OSS preparations were associated with more substantial sleep disturbances. OSS is associated with more taste intolerance, while single PEG is associated with more volume intolerance.

## Introduction

 The diagnostic accuracy and therapeutic safety of colonoscopy are highly dependent on the quality of bowel preparation. Bowel preparation is critical in determining the quality, completeness, speed, and ease of colonoscopy.^1,2^ Inadequacy of bowel cleansing is responsible for up to 33% of incomplete examinations.^3^ The efficiency of colon cancer screening by colonoscopy depends on the adenoma detection rates. Adequate bowel preparation is one of the significant determinants of adenoma detection rates and indirectly plays a crucial role in the success of a colon cancer screening program.^1^ An ideal agent should reliably empty the colon of all fecal matter rapidly without mucosal damage, patient discomfort, or fluid or electrolyte disturbances and should be inexpensive.^4,5^ Colonoscopy preparation was initially based on the principles of surgical bowel cleansing methods but has largely evolved over the last many decades in favor of osmotic laxatives.^6^ Hyperosmotic agents like mannitol, sorbitol, and lactulose were also extensively evaluated but not preferred now owing to the risk associated with combustible gas generation secondary to bacterial degradation.^7^ Sodium phosphate is well tolerated and effective in bowel cleansing; however, there are significant concerns regarding electrolyte abnormalities.^8-10^ The formulation of polyethylene glycol (PEG) by Davis and colleagues in 1980 has revolutionized bowel preparation and is currently considered the gold standard.^3,11^ Another addition to the armamentarium is the oral sulfate solution (OSS) comprising sodium sulfate, potassium sulfate, and magnesium sulfate, approved by the Food and Drug Administration of the United States in 2010 for bowel preparation.^12,13^ The mode and timing of bowel preparation were also extensively evaluated. Single-dose preparations are administered the evening before endoscopy or on the day of the procedure, preferably in the morning. In split-dose regimens, a part of the bowel preparation medication is administered on the previous evening, and the remaining medication is administered on the morning of the procedure.^14^ Published literature on the efficacy and safety of bowel preparation regimens from the Indian subcontinent is ever-increasing. The Indian diet is grossly different from that of the Western diet, and the data from the West may not be applicable in an Indian scenario.

 Within India, the staple diet and dietary contents vary significantly from state to state and may contribute to variations in results between the studies. This study was conducted in Kerala, a south Indian state, where the staple diet is rice. In contrast to the rest of India, the diet is known for its high coconut content, spices, herbs, and plentiful use of fish and vegetables. The majority of the population follows a mixed or non-vegetarian dietary pattern.^15^

## Aims

 To compare the efficacy, safety, and tolerance of split doses of OSS, split doses of PEG, and same-day single dose of PEG for bowel preparation for colonoscopy.

## Materials and Methods

 This randomized, single-blind trial was conducted at a tertiary care referral center in Kerala, India. Institutional research and ethics committee approvals were obtained for this study. Patients aged between 18 and 80 years were enrolled prospectively with informed consent and allocated based on computer-generated random allocation numbers. The inclusion criteria were (*i*) patients undergoing elective colonoscopy. Exclusion criteria were (I) age < 18 years and > 80 years, (*ii*) pregnancy, lactation, coronary artery disease, cardiac failure, liver failure, renal failure defined by an estimated glomerular filtration rate of less than 60 mL/minute, uncontrolled hypertension or diabetes mellitus, emergency colonoscopies and (*iii*) patients not willing to consent or participate in the study. Commercially available PEG powder (PEGLEC^®^; Tablets India Limited) and OSS (Coloprep^®^; Delvin Formulations Private Limited) were used for bowel preparation. Each sachet of PEG (137.15 g) contained potassium chloride 1.484 g, sodium bicarbonate 3.37 g, sodium chloride 2.93 g, sodium sulfate 11.36 g, PEG 118 g, and two optional flavor packs. Each OSS kit consisted of two bottles (177 mL each) containing sodium phosphate 17.5 g, potassium sulfate 3.13 g, and magnesium sulfate 1.6 g. All patients underwent a detailed clinical evaluation. Blood samples were collected before and after one hour of completion of the preparation agent.

 Group A (Split OSS): The first bottle was diluted with drinking water to make up to 500 mL, and it was administered from 7 PM to 8 PM on the evening before the procedure. The second bottle was prepared and administered similarly from 5 AM to 6 AM on the day of the procedure. After the contents of each bottle were administered, the patients were instructed to drink one liter of clear fluid.

 Group B (Split PEG): One sachet of PEG powder was dissolved in two liters of drinking water. Patients consumed one liter of the solution the evening before the procedure (7 PM to 8 PM) and the remaining one liter on the day of the procedure (5 AM to 6 AM). The patients were instructed to drink one liter of clear fluids after administration of the bowel preparation medication.

 Group C (same-day single-dose PEG): One sachet of PEG powder was dissolved in two liters of drinking water. On the day of the procedure, patients consumed the entire solution from 5 AM to 7 AM. After consumption, they were also instructed to drink one liter of clear fluids.

 The patients were queried regarding adverse events such as loss of sleep, nausea, vomiting, abdominal pain, and any other specific events that might have been related to the consumption of the agent. The willingness to repeat preparation, tolerance to the volume administered, and taste were also recorded. A colonoscopy was performed by any of the four experienced endoscopists blinded regarding the bowel cleansing agent administered. The quality of bowel preparation was assessed using the Boston Bowel Preparation Scale (BBPS).^16^ BBPS is a four-point scoring system applied to three segments of the colon: Right (caecum and ascending colon), transverse (transverse colon, hepatic and splenic flexures), and left (descending, sigmoid, rectum). The points are assigned as 0 - for unprepared colon with mucosa not seen due to solid fecal matter that cannot be cleared, 1- if portion of colonic mucosa is seen but other areas not well seen due to staining or residual stool or opaque fluid, 2 - if colonic mucosa is well seen but with minor amount of residual staining or small fragments of stools and or opaque fluid, 3 - if entire mucosa is well seen with no residual staining or stools or opaque fluid.^16^ Each segment received a segment score ranging from 0 to 3, and the segment scores were added together to get the overall score from 0 to 9. If the endoscopist aborted the procedure due to poor preparation, the segments proximal to that region were assigned a score of 0. The colonoscopists were well-informed regarding the use of the BBPS system. Sample images showing the grading of the bowel preparation as per BBPS were displayed in the endoscopy suites for any ready reference. Validation studies have previously revealed that a BBPS score of ≥ 5 is associated with a higher polyp detection rate and is considered adequate bowel preparation.^17^ All patients had their vitals checked at baseline, before, and after colonoscopy. Serum sodium, potassium, phosphate, and magnesium were checked in all patients before the administration of bowel preparation and 3 hours after the complete administration.

 Descriptive variables were expressed as mean ± standard deviation and compared using ANOVA and Kruskal-Wallis test. Multiple comparisons were made with the Bonferroni test. Qualitative variables were analyzed using the chi-square test. A *P* value of < 0.05 was considered significant for statistical analysis.

## Results

 A total of 333 patients were enrolled in the study; 113 received split OSS (group A), 110 split PEG (group B), and 110 single doses of PEG (group C). The mean age of patients was comparable across the groups. In groups A, B, and C, men constituted 57%, 68%, and 64%, respectively (*P* = 0.2). The indications for colonoscopy were abdominal symptoms (abdominal pain, diarrhea, constipation, haematochezia) to assess the disease extent or treatment response in patients with inflammatory bowel disease or ileocecal tuberculosis and for screening or surveillance of colorectal neoplasms. Baseline variables are shown in Table 1.

**Table 1:** Baseline characteristics enrolled subjects

**Parameter**	**A**	**B**	**C**	***P***
Age (y)	45.7 ± 19.2	47.8 ± 18.4	46.2 ± 15.4	0.65
Sex (% M: F)	57%: 43%	68%: 32%	64%: 36%	0.2
S. sodium (mEq/L)	136 ± 5.05	136 ± 4.85	136 ± 5.24	0.9
S. potassium (mEq/L)	4.13 ± 0.6	4.04 ± 0.53	4.09 ± 0.56	0.48
S. calcium (mg/dL)	9.29 ± 0.91	9.33 ± 0.74	9.38 ± 0.75	0.7
S. magnesium (mg/dL)	1.94 ± 0.33	1.94 ± 0.26	1.9 ± 0.28	0.5

Parametric variables are expressed as (Mean ± SD).

###  Efficacy of Colon Cleansing 

 The mean total BBPS scores were 8.08, 7.52, and 7.92 with regimens A, B, and C, respectively (*P* = 0.076). Segmental preparation qualities were statistically similar in the right and transverse segments of the colon across different groups but significantly different in the left colon. Post hoc Bonferroni analysis for BBPS of the left colon revealed a significant difference between group A versus B (2.79 versus 2.54; *P* = 0.008) and between group C versus B (2.75 versus 2.54; *P* = 0.031). The mean time to reach the caecum was also similar between the groups. Ileal intubation rates were 46%, 50%, and 40.1% in groups A, B, and C, respectively (*P* = 0.4). Efficacy-related parameters are summarized in Table 2.

**Table 2:** Comparison of the BBPS scores, total and segmental, and colonoscopy indices achieved with the bowel preparation regimens

**Parameter**	**A**	**B**	**C**	***P***
BBPS left colon	2.79 ± 0.49	2.54 ± 0.71	2.75 ± 0.57	< 0.01
BBPS transverse colon	2.69 ± 0.56	2.50 ± 0.77	2.67 ± 0.64	0.075
BBPS right colon	2.62 ± 0.64	2.49 ± 0.76	2.54 ± 0.68	0.45
BBPS total score	8.08 ± 1.48	7.52 ± 2.10	7.92 ± 1.74	0.076
Time to reach caecum (min)	18.2 ± 13.2	18 ± 11.3	16.4 ± 10.2	0.51
Ileal intubation rates (n; %)	52; 46%	55; 50%	45; 41%	0.4

Parametric variables are expressed as (Mean ± SD).

###  Safety and Tolerance

 The adverse events noted in our study, such as nausea, vomiting, and abdominal pain, were similar across the three agents. 36 (31.86%) patients who had consumed OSS reported severe taste intolerance; however, the same was reported only by seven (6.37%) and nine (8.18%) patients in groups B and C, respectively. Split OSS and PEG regimens were associated with sleep disturbances in 57 (50%) and 56 (51%), respectively, while same-day single-dose PEG was not associated with sleep disturbances. Volume intolerance was reported more with single-dose PEG (59%) compared with split PEG (51%) and split OSS (26%) regimens. The mean change in serum sodium, potassium, calcium, and magnesium concentrations with preparation was similar to the agents. Table 3 summarizes the safety and tolerance parameters for the three regimens.

**Table 3:** Comparison of the patient-reported intolerances and electrolyte deviations with the bowel preparation regimens

**Parameter**	**A**	**B**	**C**	***P***
Nausea	37 (33%)	28 (25%)	23 (21%)	0.129
Vomiting	16 (14%)	24 (22%)	14 (13%)	0.14
Abdominal pain/cramps	28 (25%)	22 (20%)	17 (15%)	0.22
Taste intolerance	99 (88%)	80 (73%)	78 (71%)	< 0.01
Volume intolerance	29 (26%)	56 (51%)	65 (59%)	< 0.001
Sleep disturbance	57 (50%)	56 (51%)	0 (0%)	< 0.001
Willingness to repeat	108 (96%)	105 (95%)	107 (97%)	0.83
Change in sodium	-1.03 ± 4.6	-0.6 ± 4.81	-0.85 ± 4.36	0.783
Change in potassium	-0.02 ± 0.55	0.01 ± 0.51	0.03 ± 0.5	0.702
Change in calcium	-0.00 ± 0.65	0.05 ± 0.62	0.03 ± 0.50	0.868
Change in magnesium	-0.04 ± 0.31	0.01 ± 0.33	-0.04 ± 0.31	0.498

Non-parametric variables are expressed as the number of patients (percentage). Parametric variables are expressed as mean ± SD.

###  Inpatient Versus Outpatient Bowel Preparation 

 A total of 211 patients underwent bowel preparation as inpatients, while 122 were prepared as outpatients. The mean ( ± SD) total, left, transverse, and right BBPS scores for inpatients were 7.75 ( ± 1.85), 2.66 ( ± 0.63), 2.59 ( ± 0.67) and 2.52 ( ± 0.70), respectively while the scores for outpatients were 7.98 ( ± 1.71), 2.74 (0.56), 2.67 ( ± 0.64) and 2.60 ( ± 0.69). There was no statistical difference in the total or segmental scores between the inpatient and outpatient groups (*P* = 0.279, 0.246, 0.303, and 0.361).

###  Duration Between Complete Consumption of Preparation Agent and Initiation of Colonoscopy

**Figure 1:**
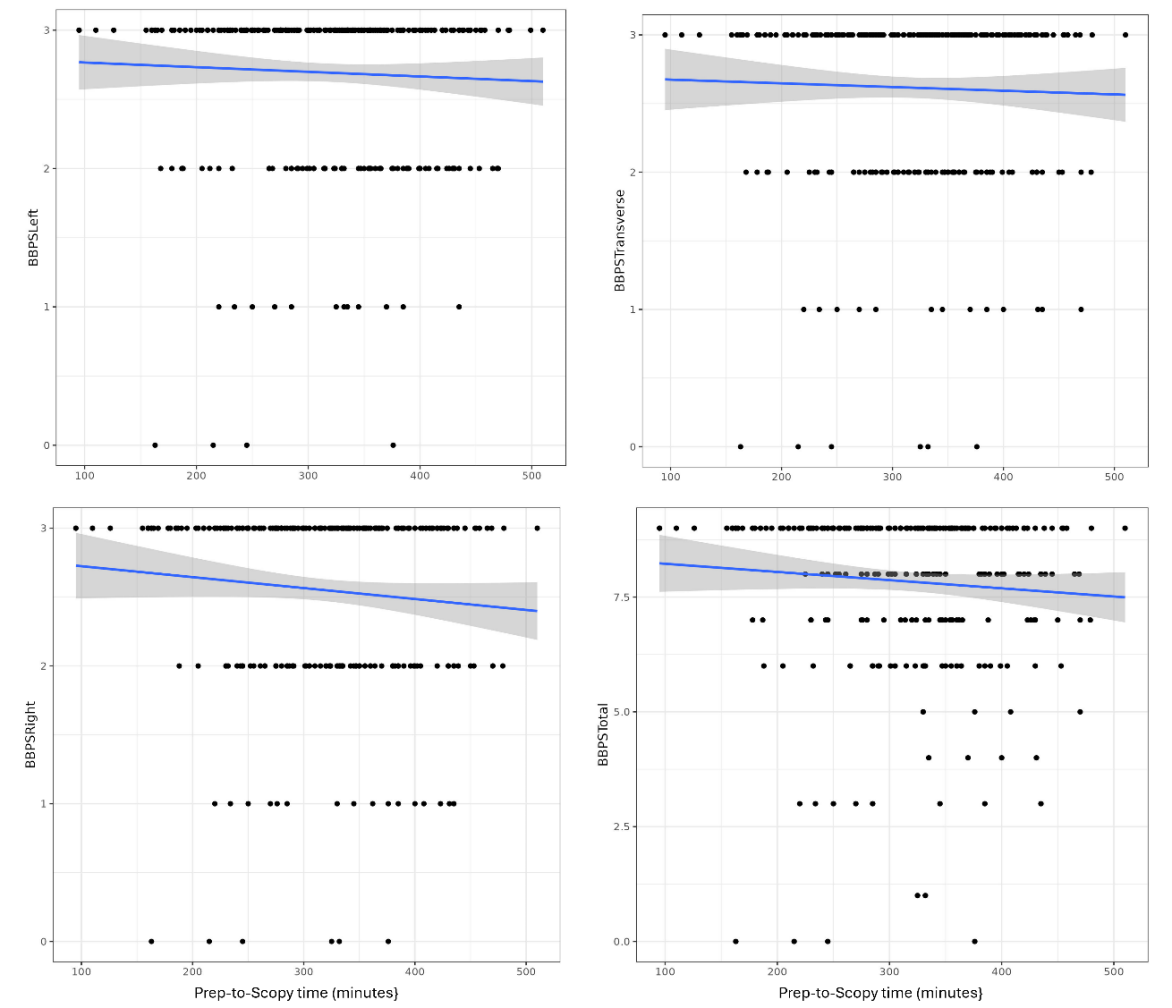
Linear regression plots depicting the distribution of BBPS scores depending on the time interval between preparation and colonoscopy

 The mean time interval between administration of the preparation and colonoscopy was 317 ( ± 79.2) minutes. The quality of preparation tended to decline over time, as depicted in linear regression plots (Figure 1). Based on the time interval between the complete consumption of the preparation agent and the initiation of colonoscopy, patients were classified into three groups: less than 4 hours, 4 to 6 hours, and more than 6 hours. Subgroup analysis did not reveal a statistically significant difference in mean BBPS scores (Table 4).

**Table 4:** Analysis of the quality of bowel preparation with time interval between preparation and colonoscopy

	**<4 hours**	**4-6 hours**	**>6 hours**	***P***
BBPS left colon	2.65 ± 0.74	2.74 ± 0.56	2.62 ± 0.60	0.232
BBPS transverse colon	2.60 ± 0.77	2.63 ± 0.64	2.60 ± 0.66	0.904
BBPS right colon	2.67 ± 0.77	2.56 ± 0.66	2.48 ± 0.71	0.320
BBPS total score	7.93 ± 2.27	7.91 ± 1.70	7.66 ± 1.72	0.520

## Discussion

 Colonoscopy in a patient with an unprepared or incompletely prepared colon can result in missing lesions and also can lead to complications.^1-5^ This emphasizes the role of good bowel preparation before colonoscopy. Preparations have considerably evolved over many decades; however, an ideal agent remains a myth. Although medical literature search provides a large body of evidence on bowel preparation agents, their usage, efficacy, tolerance, and adverse effects, there is a dearth of literature from the Indian subcontinent. Indian diet is grossly different from the Western diet due to higher fiber content; hence, the Western data may not always be applicable in our region.^18^ The lower educational status may also interfere with understanding the administration of bowel preparation agents. Limited access to toilet facilities and drinking water can also influence the quality of bowel preparation in this part of the world. Hence, regional data on the efficacy and tolerability of different bowel preparation regimens was required, and the current study was performed with this intention.

###  Efficacy of Colon Cleansing

 The current study revealed no statistically significant difference in the total mean BBPS scores between the three regimens. The scores were marginally higher in the split OSS and single PEG arms when compared with the split PEG arm, but the difference was statistically insignificant. Analysis of the segmental scores of the right and transverse colon revealed a similar pattern. However, analysis of the segmental BBPS score of the left colon showed split PEG to be significantly inferior to single PEG and split OSS preparations.

 Studies suggest that bowel cleansing quality with split PEG is equal to or superior to single PEG.^19-25^ However, most of these studies compared overnight split PEG (2L + 2L) versus previous day single dose PEG (4L). Seo and colleagues compared same-day 2L PEG with split PEG (2L + 2L) and found similar efficacy in bowel cleansing. However, the low-volume PEG patients were put on dietary restrictions while the split group continued on the standard diet.^26^ Our study failed to demonstrate statistical superiority for split or single PEG in total BBPS scores. However, the segmental BBPS scores for the left colon were superior for the single PEG regimen. The differences in the volume of PEG for single and split preparations, non-concomitant usage of any other laxatives, same-day use of PEG for single-dose preparation, no specific dietary restrictions, and the differences in the South Indian diet, especially the high fiber and medium chain triglyceride content in the diet all might have resulted in the differences in our results when compared with Western literature.

 OSS, unlike PEG, has undergone less extensive evaluation. Split OSS provided excellent preparation more frequently compared with the 4L single dose PEG (71.4% vs. 34.3%) in the study by Rex et al.^12^ The study by Di Palma et al split OSS is associated with more excellent preparations than split PEG (63.3% vs. 52.5%).^13^ Nam and colleagues concluded that OSS was more efficient for bowel preparation than PEG-ascorbic acid, especially in elderly and female patients.^27^ A meta-analysis of seven studies comparing OSS and low-volume PEG-ascorbic solution, including 2049 patients, published in 2022, demonstrated comparable adequate bowel preparation rates and a marginally higher chance of excellent bowel preparation with OSS; however, it was associated with a greater intolerance.^28^ The current study found no statistically significant differences in the overall quality of bowel preparation with split OSS compared to split PEG and single PEG. However, the left colonic BBPS scores were superior for split OSS compared with split PEG, and the left colonic scores were statistically comparable between split OSS and single PEG.

###  Safety and Tolerance

 The incidence of nausea, vomiting, abdominal pain or discomfort, changes in electrolyte levels baseline, and the willingness to repeat preparation if required were not different between the three study groups. Sleep disturbances were more familiar with overnight split regimens of OSS and PEG. As the single PEG regimen was administered in the current study only on the morning of the colonoscopy, it was not associated with any sleep disturbances. Taste intolerance was significantly higher with OSS, while patients with single PEG reported volume intolerance significantly more than split PEG or OSS. Studies have demonstrated similar adverse effect profiles, compliance, and overall tolerance for split and single PEGs.^21,24,29^ Studies have also shown fewer sleep disturbances with same-day morning dose PEG than overnight split PEG or day-before procedure single dose PEG.^26,30^ Published trials evaluating OSS have demonstrated similar adverse effect profiles between split OSS, single PEG, and split PEG.^12,13^ Available literature also reports greater ease in completing the split-dose preparations than single-dose administration.^31^ Sulfate moiety is known to impart a lousy taste to fluids. Sulfate-free PEG was introduced into the market to overcome the poor taste of PEG preparations. Sulfate is a significant component of OSS, and poor taste is the major drawback of this preparation.^32^ No significant complications were associated with any of the bowel preparations. Despite being a hyperosmotic agent, OSS use was not associated with substantial electrolyte disturbances.

###  Inpatient Versus Outpatient Bowel Preparation 

 The current study revealed no significant difference in the quality of bowel preparation between inpatients and outpatients. Previous studies have demonstrated a superior bowel cleansing for ambulatory patients rather than for hospitalized patients.^33-35^ The proposed reasons for poor quality preparation in hospitalized patients include associated co-morbidities for which they are admitted, use of medications with constipating effect, comparatively restricted access to drinking water and toilets, being less ambulant and delay in access to preparation agents owing to the busy hospital environment.^35,36^ The current study did not include patients with significant co-morbid illnesses; most of our patients were ambulant. All patients were thoroughly instructed regarding the preparation regimen by gastroenterology residents. These might have contributed to the similarity in the quality of bowel preparations between inpatients and outpatients. Janahiraman and colleagues demonstrated improved quality of bowel preparation for colonoscopy with better patient education.^37^

###  Effect of Preparation-to-Colonoscopy Interval

 The current study failed to reveal any statistically significant difference in the quality of bowel preparation segment-wise or total with a time interval to the initiation of colonoscopy. Several studies have evaluated the impact of the time interval between the completion of bowel preparation and initiation of colonoscopy on the quality of bowel preparation in the past.^36^ A study by Seo et al revealed the interval between the last dose of PEG and the initiation of colonoscopy is an important factor in determining bowel preparation. They concluded that 3 to 5 hours intervals provided the most optimal results.^17^ This study was a non-randomized trial using only a split PEG regimen and Ottawa scale for assessment of the quality of bowel preparation. A study by Eun et al found that an interval of 4 hours or less between the end of PEG intake and the start of colonoscopy is superior to those with intervals of more than 4 hours.^38^ Dallas Veteran’s Affairs Medical Center study concluded that colonoscopy should begin 14 hours after preparation to avoid unsatisfactory bowel preparations.^39^ In our study, patients who underwent colonoscopy within the first 6 hours of completion of bowel preparation had a marginally superior bowel preparation compared with more than 6-hour intervals, but the difference was statistically insignificant. The vast majority of our patients had colonoscopies done between 4 to 6 hours of completion of bowel preparation, while the number of patients in the less than 4 hours and more than 6 hours were comparatively less. The differences in study protocol, use of multiple colonoscopy preparation regimens, differences in the assessment of preparation quality, and the uneven distribution of different time frames might have contributed to any deviation of our results from the published data.

 The significant advantage of this study is its randomized endoscopist-blinded design with a relatively large number of patients. Our study also has tried to evaluate different aspects of colonoscopy preparation, including efficacy, tolerance, adverse effects, electrolyte disturbances, patient status, whether inpatient or not, and the time interval between the end of preparation and initiation of colonoscopy. Even though the colonoscopies were performed by experienced gastroenterologists who are well aware of the BBPS, there is always the likelihood of inter-observer variability, which constitutes this study’s major drawback.

## Conclusion

 Split OSS, split PEG, and single PEG regimens provide adequate and comparable bowel preparation for colonoscopy with good patient tolerance and no significant adverse effects. Overnight PEG and OSS preparations are associated with more significant sleep disturbance. OSS is associated with more taste intolerance, while single PEG is associated with more volume intolerance.
